# Learning in the Lassa Belt

**DOI:** 10.4269/ajtmh.18-0287

**Published:** 2018-11

**Authors:** Heidi Sandige

**Affiliations:** Phebe Hospital, Global Health Service Partnerships, Suakoko, Bong County, Liberia

Bong County Liberia sits directly in the center of the Lassa fever belt. Only a few years ago, Ebola fever was rampaging here, so hemorrhagic fevers raise appropriate alarm in health-care workers. Clinicians from this facility lost their lives during that time. Health-care workers infrequently wash their hands (we have buckets with taps but no running water, and hand sanitizer is rare), but do not touch patients without gloves. Unfortunately, all sorts of things are handled without gloves—bedding and charts and staff lunches eaten communally at the nurses’ stations. The hospital is perpetually short of gloves. We have on-site Ebola tests, but Lassa fever testing is sent out to Sierra Leone.

I am here serving as a pediatric physician educator, and it is my first time doing clinical work in the Lassa fever belt. My trainees are mostly from the city, so even though they are Liberian, Lassa fever is often new to them as well. We are working through this together. Most of them were here for Ebola. I was not.

The agency for which I work requests that physician educators not see patients with hemorrhagic fevers. However, patients do not walk in the door with their diagnoses stamped on their foreheads. I cannot refuse to see patients with Lassa fever because I do not know they have Lassa fever until I see them, and I frequently do not know until quite a while after that. The World Health Organization case definition for Lassa fever includes 48 hours of no clinical response to antimalarial or antibiotic treatment.

My first patient with Lassa fever presented with what looked like typhoid. Five-year-old Sara had abdominal pain, diarrhea, fever, and rigors. I observed the chills and rigors, and because Sara stayed conscious and answering questions throughout, I did not classify the episode as seizures. In this setting, health-care workers rarely distinguish between different kinds of “jerking.” This is a serious shortcoming because tetanus is rampant here as well, in the same populations that have cerebral malaria and chill-inducing illnesses. Sara had a story consistent with typhoid, and a negative malaria smear. Although Widal testing is unreliable, it is one test that is regularly performed (a blood count is not available), so strongly positive H and O titers steered us toward typhoid.

Sara improved somewhat with fluids and Ciprofloxacin. The rigors disappeared and were not replaced by true seizures, but she continued to have fever, abdominal pain, vomiting, and diarrhea. The emesis was copious and violent, as her family tried to keep her hydrated. We suspected there might be some partially digested blood in the emesis, and we worried about intestinal perforation, which does sometimes occur. We changed her antibiotics when the fevers would not resolve and worried that the facial and cervical swelling—soft rather than matted—could be from subcutaneous emphysema from the violent emesis. At about this time, a local colleague explained to me that this swelling was strongly suggestive of Lassa fever. Sara was swept away into isolation for testing and presumptive treatment. She was positive for Lassa fever and did well. The presentation of a soft, full neck and face became a trigger for me, one I had not previously associated with Lassa fever.

We continued to have Lassa-positive patients, sometimes several in a week, of various ages. There are deaths in the emergency room and in the isolation unit. Edema of the neck is occasionally present. A urinalysis positive for blood is frequently the trigger for isolation and presumptive treatment, but patients in our population also sometimes have schistosomiasis, which is associated with hematuria but not conjunctivitis. The presentation most suspicious of Lassa fever at our hospital tends to be a patient who presents with conjunctivitis, fever, and excessive vomiting. In any season, the vast majority of pediatric patients here are admitted with the diagnosis of “severe malaria and anemia.” Often, this proves to be correct.

When another young girl, Quita, was admitted to the pediatric intensive care unit from the emergency room with the story of high fevers and jerking, she was diagnosed with presumed cerebral malaria. Her malaria smear was positive with high parasitemia, and her hematocrit was low. It was difficult to elucidate a good history, but in this case, the grandmother admitted to having poured various “country medicines” (herbal concoctions) down the child’s nose and mouth in the last few days. When I first saw Quita, she had a gauze-wrapped tongue depressor in her mouth, soaked in blood, and the nurses explained that she had bitten her tongue during a seizure. She had clearly depressed mental status, responsive to voice and pain but in no way appropriate or oriented.

If Quita truly had bitten her tongue to the degree that she was continuing to seep blood several hours later, I thought I might need to repair that laceration. I used gloves and a mask because I was getting right up into her face, and used the rarely used otoscope to take a peek in her nostrils where I saw some friable and ulcerating but not hemorrhaging lesions that I suspected might be the damage inflicted by pouring hot liquid in her nose. I removed the tongue depressor and the blood clots from her mouth, but found no lacerations—only more rounded lesions on the mucous membranes of her mouth: dental ridges, upper posterior palate, and buccal surfaces. A very small number of these were oozing, but most of them did not appear to be actively bleeding. We covered her for bacterial sepsis and cerebral malaria.

Later that afternoon, Quita went into respiratory distress and began to bleed more copiously from the lesions in her mouth. We tried nasal cannula and face mask support, but eventually also had to insert an oral airway and repeatedly clear the blood from her mouth. She received short-term manual positive pressure ventilation more than once, but as there are no ventilators in our institution, intubation was not an option. We ordered fluids and blood for her evolving shock. Certainly, at this point, we strongly suspected Lassa fever, and ordered Lassa (and Ebola) testing and isolation, but she died before the positive result was available or the transfer could be completed.

Both children spent more than 12 hours in our regular wards, where beds are not quite three feet apart and caregiving parents share space throughout. Quita’s case raised more alarm because of the bleeding and the clearly bloody resuscitation. Some patients do present with or develop the hemorrhagic symptoms—an IV that bleeds or a random glucose measurement prick that will not clot. Infants with these symptoms frequently die before testing. Although we are almost certainly missing a large number of cases, no set of symptoms is specific to Lassa fever. Cerebral malaria is more common. Cough and retrosternal pain are associated symptoms, but our hospital treats many cases of respiratory illness each day, without a strong suspicion of Lassa.

I was recently called to the ER to evaluate a 20-month-old child, Flomo, because the clinician got a history of recent antibiotic exposure and suspected Stevens–Johnson syndrome. Flomo had stomatitis and coalescing full thickness erosions of the oral mucosa, but a diaper rash of a different character that did not extend to the mucous membranes. He also had edema of the head and neck and developed convulsions shortly after I evaluated him. I ordered Lassa fever testing, isolation, and antiviral treatment according to the hospital protocol. This child did not receive testing before his death. It is not always clear when Lassa fever should be suspected. It is frustrating to us all when we have no answer, no definitive explanation.

One of the dedicated emergency nurses here was diagnosed with Lassa fever, possibly because of exposure to Flomo. He did not comply with therapy, left the isolation unit, and died “up country” (not at a hospital). This sequence of events is hauntingly like the beginning of the Ebola outbreak. As far as I know, nobody touched this child without gloves. Workers almost certainly touched the bed, the drapes, the nasal cannula, and the lapa (cloth) on which he lay. For the second time this year, I am texting my temperature into my program director every morning.

Lassa fever testing is coming to our hospital. A colleague and I have printed up Lassa fever treatment protocols. Staff meetings frequently highlight the glove shortage; the deficiency in universal precautions receives less attention and deserves more. Developing a system for identifying Lassa fever is proving difficult; so many endemic syndromes have overlapping clinical presentations. In the meantime, we are just learning in the Lassa belt.

